# Women Undergoing Hormonal Treatments for Infertility: A Systematic Review on Psychopathology and Newly Diagnosed Mood and Psychotic Disorders

**DOI:** 10.3389/fpsyt.2020.00479

**Published:** 2020-05-26

**Authors:** Alexandre González-Rodríguez, Jesús Cobo, Virginia Soria, Judith Usall, Clemente Garcia-Rizo, Miquel Bioque, José Antonio Monreal, Javier Labad

**Affiliations:** ^1^ Department of Mental Health, Parc Tauli Hospital Universitari, Institut d’Investigació i Innovació Parc Tauli (I3PT), Autonomous University of Barcelona (UAB), Centro de Investigación Biomédica en Red en Salud Mental (CIBERSAM), Sabadell, Spain; ^2^ Department of Psychiatry, Bellvitge University Hospital, Bellvitge Biomedical Research Institute (IDIBELL), Department of Clinical Sciences, University of Barcelona (UB), Centro de Investigación Biomédica en Red en Salud Mental (CIBERSAM), Hospitalet de Llobregat, Barcelona, Spain; ^3^ Mental Health Services, Parc Sanitari Sant Joan de Déu, Sant Boi de Llobregat, Spain; ^4^ Barcelona Clinic Schizophrenia Unit, Neuroscience Institute, Hospital Clinic de Barcelona, Institut d’Investigacions Biomèdiques August Pi i Sunyer (IDIBAPS), Centro de Investigación Biomédica en Red en Salud Mental (CIBERSAM), Department of Medicine, University of Barcelona, Barcelona, Spain

**Keywords:** infertility, sex hormones, fertility treatments, psychosis, affective

## Abstract

**Background:**

The association between infertility treatments and mental disorders has been poorly addressed. This work aims to review current evidence on the psychopathological effects of hormonal treatments used for infertility on women and the occurrence of newly diagnosed mood and psychotic disorders.

**Methods:**

A systematic review was performed by searching PubMed and clinicaltrials.gov databases from inception until September 2019. Clinical trials on hormone treatments for infertility in patients with mood or psychotic disorders, as well as those evaluating the onset of symptoms, were included. Selected studies were published in English, Spanish, and Dutch language peer-reviewed journals. Preferred Reporting Items for Systematic Reviews and Meta-Analyses (PRISMA) guidelines were followed. Observational studies and case reports were excluded. Effect sizes for changes in depressive symptoms were calculated with Hedges’g and Cohen’s d confidence intervals. A meta-analysis was not performed due to the heterogeneity of hormonal compounds in protocols.

**Results:**

From 1,281 retrieved records, nine trials were included; all of them were conducted in non-clinical populations. Four trials compared Gonadotropin-releasing hormone (GnRH) agonists and GnRH antagonists, showing a better mood profile for hormonal protocols including antagonists in one trial. Two trials compared protocols using GnRH agonists/antagonists versus natural cycle protocols (without gonadotropin stimulation), with a better mood profile (less depressive symptoms) in those protocols without gonadotropin stimulation. Other studies compared long and short protocols of GnRH agonists (no differences); two GnRH agonists, buserelin, and goserelin (no differences); and two patterns of clomiphene vs placebo administration (no differences). None of the selected studies investigated the risk of relapse in women with a previous diagnosis of depressive or psychotic disorders. When exploring pre-post changes in depressive symptoms, effect sizes suggested mild mood worsenings for most protocols (effect sizes ≤ -0.4), with the following pattern (worse to better): GnRH agonist > GnRH antagonist > no gonadotropin stimulation.

**Conclusions:**

This is the first systematic review exploring the psychopathological effects of hormonal infertility treatments. Our study suggests that protocols without gonadotropin stimulation show a better mood profile when compared to those using GnRH antagonists or GnRH agonists. Future studies need to include patients with major mood and psychotic disorders.

## Introduction

Women with fertility problems have been extensively found to suffer from psychological burden and may experience this clinical situation as very stressful (1). The relationship between assisted reproductive technologies (ARTs) and mental distress is complex, and it calls for a careful examination of the direction of the effects between both variables (2–4).

ART treatments may increase mental distress in women undergoing these therapeutic options (1). Mental health consequences may be partially explained by psychological factors, such as neuroticism, as well as by biological factors derived from the biochemical nature of treatment compounds (3).

When focusing on reproductive medicine, it seems to be crucial to determine whether a woman is infertile (5). Medical history (e.g., health history, the use of other medications); physical examination; blood tests including assessments of hormone levels; and ultrasonography exploring the ovaries, uterus and Fallopian tubes appear to be mandatory (5, 6). In some cases, infertility in women might be secondary to an ovulation problem or an obstruction of the Fallopian tubes (7). However, in 5%–15% of cases, all tests and physical examinations are normal, and specific causes cannot be determined (8).

Once the cause of infertility is found, all potential options for treatment should be considered (9). Briefly, four main types of infertility treatment are available: gamete intrafallopian transfer (GIFT) and zygote intrafallopian transfer (ZIFT), intrauterine insemination (IUI), and *in vitro* fertilization (IVF) techniques (10). IUI is a fertility treatment based on placing sperm directly into the uterus while the woman is ovulating (11). IVF is an ART process based on controlled ovarian stimulation, egg retrieval from women’s ovaries and the fertilization of these eggs with sperm in the laboratory (11). Finally, the embryo is placed in the woman’s uterus. GIFT and ZIFT refer to the collection and placement of gametes or zygotes, respectively, into the Fallopian tube (12).

Regarding IVF techniques, we will focus on the use of biological compounds, mainly hormones, aiming to achieve controlled ovarian stimulation. These molecules are factors that can be associated with psychopathological changes in healthy women and women with previous mental disorders (9).

Many biological compounds have been developed for ovulation induction in IVF, with the main aim of obtaining more oocytes (13), including clomiphene citrate, aromatase inhibitors, gonadotrophins, and gonadotropin-releasing hormone (GnRH) analogues (9). Clomiphene citrate blocks oestrogen receptors and increases follicle-stimulating hormone (FSH) levels. Aromatase inhibitors block the conversion of androgens to oestrogens. Gonadotrophins include recombinant FSH and luteinizing hormone (LH), and GnRH analogues include agonists and antagonists.

Conventional controlled ovarian stimulation protocols include the administration of gonadotropin-releasing hormone (GnRH) agonists with the main aim of the desensitization of the pituitary gland through the suppression of the release of both pituitary follicle-stimulating hormone (FSH) and luteinizing hormone (LH) (13). This technique has been successfully associated with IVF; however, several adverse effects have also been described, such as mood disturbances (3). More recently, GnRH antagonist protocols have been found to be an alternative for ovarian stimulation. They seem to show lower rates of ovarian hyperstimulation syndrome and may reduce the length of ovulatory stimuli compared with other ovulation induction protocols (14). GnRH antagonist protocols are based on the daily administration of recombinant follicle stimulating hormone (r-FSH) analogue to start ovarian stimulation (2–3 cycle days). Some studies have found GnRH protocols to have higher rates of pregnancies than treatment with clomiphene or aromatase inhibitors (13). More recently, a short GnRH antagonist protocol has been recommended for younger women undergoing their first ART cycle (14).

Nevertheless, sex hormones have been found to regulate mood and may play an important role on the pathophysiology of affective disorders as well as schizophrenia and other psychotic disorders (15). Rubinow and Schmidt proposed several models to explain how sex may influence brain function by means of hormonal effects and how the regulation of affect may be sex dependent (16). These investigations have also been conducted in patients with psychotic disorders. If the response to stress may be dysregulated in psychosis, Goldstein and co-workers (17) reported that the interplay between steroid hormones and neural activity may be sex dependent in psychosis, particularly for brain functions implicating the prefrontal cortex (17, 18).

In the particular case of GnRH agonists, it should be noted that they combine hormonal control of the cycle by means of a hypoestrogenism state. This short induced-hormonal state may determine subsequent mental health consequences that may be partially explained through the hypothalamic-pituitary-gonadal axis (19). Hormonal changes and withdrawal of oestrogens may determine an increased vulnerability to depression in women as well as a poorer prognosis (worsening of psychotic symptoms, greater risk of relapse and higher needs of antipsychotic dosages) in female patients with schizophrenia (20, 21). Therefore, those fertility treatments that induce hypoestrogenism may have a negative effect on depressive or psychotic symptoms.

Although some studies have addressed the effects of sex steroids in brain functions in individuals with and without mental illnesses (15–18), no previous systematic reviews have explored the effects of infertility hormonal treatments on psychopathology in women. The vast majority of the studies have investigated pregnancy outcomes or other somatic concerns, and psychopathological symptoms have been widely neglected.

Several observational studies have investigated the prevalence rates for psychiatric disorders in women undergoing fertility treatments (22). The authors found a prevalence of mood disorders around 26% in women, highlighting that major depression was the most common diagnosis. In contrast with these findings, Salih Joelsson et al. (23) compared depressive symptoms in pregnant and non-pregnant women, and pregnant women receiving fertility treatment (23). Pregnant women did not differ in depressive symptoms compared to the other two groups, suggesting a lack of negative effect of fertility treatments on psychopathological symptoms. More recently, Freeman et al. (24) carried out a prospective observational study in women with a previous history of unipolar or bipolar depression undergoing fertility treatment (24). They concluded that maintenance of psychotropic medication was not sufficient for avoiding affective recurrences. With regard to schizophrenia and other psychoses, a national register study compared the success rates of fertility in women with and without psychotic disorders (25), although exacerbation of psychotic symptoms was not investigated.

In brief, findings from cohort and observational studies seem to be contradictory and inconclusive. Therefore, we aimed to systematically review current evidence on the psychopathological effects of hormonal treatments for infertility on women and the influence of these treatments on the occurrence of newly diagnosed mental disorders. We focused mainly on investigations concerning women with mood disorders and psychotic disorders.

## Methods

### Search Strategy

A systematic computerized search was performed by focusing on trials evaluating the effect of hormone treatments for infertility in major depression, bipolar disorder, schizophrenia, and other psychotic disorders. PubMed database and ClinicalTrials.gov were searched from inception until September 2019 in keeping with the Preferred Reporting Items for Systematic Reviews and Meta-analyses (PRISMA) guidelines (26). Electronic search was also completed by revising all references of including studies for potential papers to be included.

The following search terms were used: (clomiphene OR GnRH OR letrozole OR vorozole OR anastrozole OR aromatase inhibitors OR FSH OR LH OR r-FSH OR r-LH OR *in vitro* fertilization OR IVF OR ICSI OR intracytoplasmic sperm injection OR ovarian stimulation OR controlled ovarian hyperstimulation) AND (psychiatric OR psychopathology OR psychopathological OR relapse OR recurrence OR depression OR bipolar OR mania OR psychosis OR schizophrenia) AND trial.

### Inclusion Criteria

Studies that met the following inclusion criteria were considered: a) trials focused on evaluating the effect of hormonal treatment for infertility on women; b) studies that evaluated psychopathological effects of infertility treatments on women without a diagnosis of mental disorders (newly diagnosed mental disorder) or in women with a previous mental disorder (major depression, bipolar disorder, schizophrenia and other psychotic disorders); c) published trials in peer-reviewed journals or registered in clinicaltrials.gov; d) articles written in English, Spanish or Dutch language; and e) studies that assessed the effectiveness of hormone compounds for the treatment of infertility.

The exclusion criteria were as follows: a) naturalistic studies and case reports, b) studies assessing the effect of hormone compounds administered for uses other than the treatment of infertility, and c) studies exploring the effectiveness of hormone drugs to treat mental disorders.

### Data Collection and Extraction

Titles and abstracts of studies identified in the initial searches were screened independently by two review authors (AG-R and JC). The same authors extracted data independently. Any disagreements or discrepancies between them were resolved by consensus and were explored with a third additional reviewer. Full-text documents were also reviewed. From the initially selected articles, those that did not meet our inclusion criteria or met any exclusion criterion were excluded. The last search was conducted on 29th October 2019. The PICO method for the systematic review is detailed in **Table S1**.

### Risk of Bias

The risk of bias was evaluated using the Cochrane risk of bias tool. Studies were classified into three categories attending to their quality: good, fair, and poor. A meta-analysis was not performed due to the heterogeneity of the hormonal treatment protocols.

### Effect Sizes for Changes in Depressive Symptoms

For those clinical trials that included information on depressive symptoms at baseline and after the hormonal treatment for infertility, effect sizes were calculated. Hedges’ g was used as a measure of the effect size for changes in depressive symptoms after the hormonal protocol using an on-line calculator that allows the calculation of the effect size for paired samples tests (https://effect-size-calculator.herokuapp.com/#paired-samples-t-test). Another effect size measure considered in the systematic review was confidence intervals (lower and upper limits) using Cohen’s d. These measures were calculated using pre-post scores (depressive symptoms) and standard deviation measures of all studies with available data. If data were available as the median (interquartile range), the mean and SD were estimated as described previously (27). In a few cases in which data were only available in figures, we extracted this information from figures using the same procedure explained in a recent systematic review (28). Previous studies suggest that data extraction from figures shows high precision and seems to be a useful option to perform a meta-analysis when randomized clinical trials publish figures as the only source of outcome data (29).

Because the correlation between pre- and post-scores is required to impute the standard deviation within groups from the standard deviation of the difference, this correlation needs to be known for calculating the confidence intervals in effect sizes (30). As these correlations were not reported in studies, we calculated different confidence intervals using different estimated correlations (r = 0, r = 0.25, r = 0.5, r = 0.75). The confidence intervals calculated with a correlation of 0.5 will be included in a table in the article and the confidence intervals calculated with different correlations (sensitivity analyses) will be included in the supplementary material (**Table S3**).

Hedges’g and Cohen’s d are effect size measures that indicate the standardized difference between two means. It is assumed that 0.2 can be considered a “small” effect size, 0.5 represents a “medium” effect size and 0.8 a “large” effect size (31). In our systematic review, as effect sizes represent changes in depressive symptoms, positive effect sizes would imply mood improvement (reduction in depressive scores after hormonal treatment) whereas negative effect sizes would indicate mood worsening. Effect sizes closer to zero correspond to hormonal treatments that were relatively “neutral” in terms of changing the mood status.

## Results

**Figure 1:**
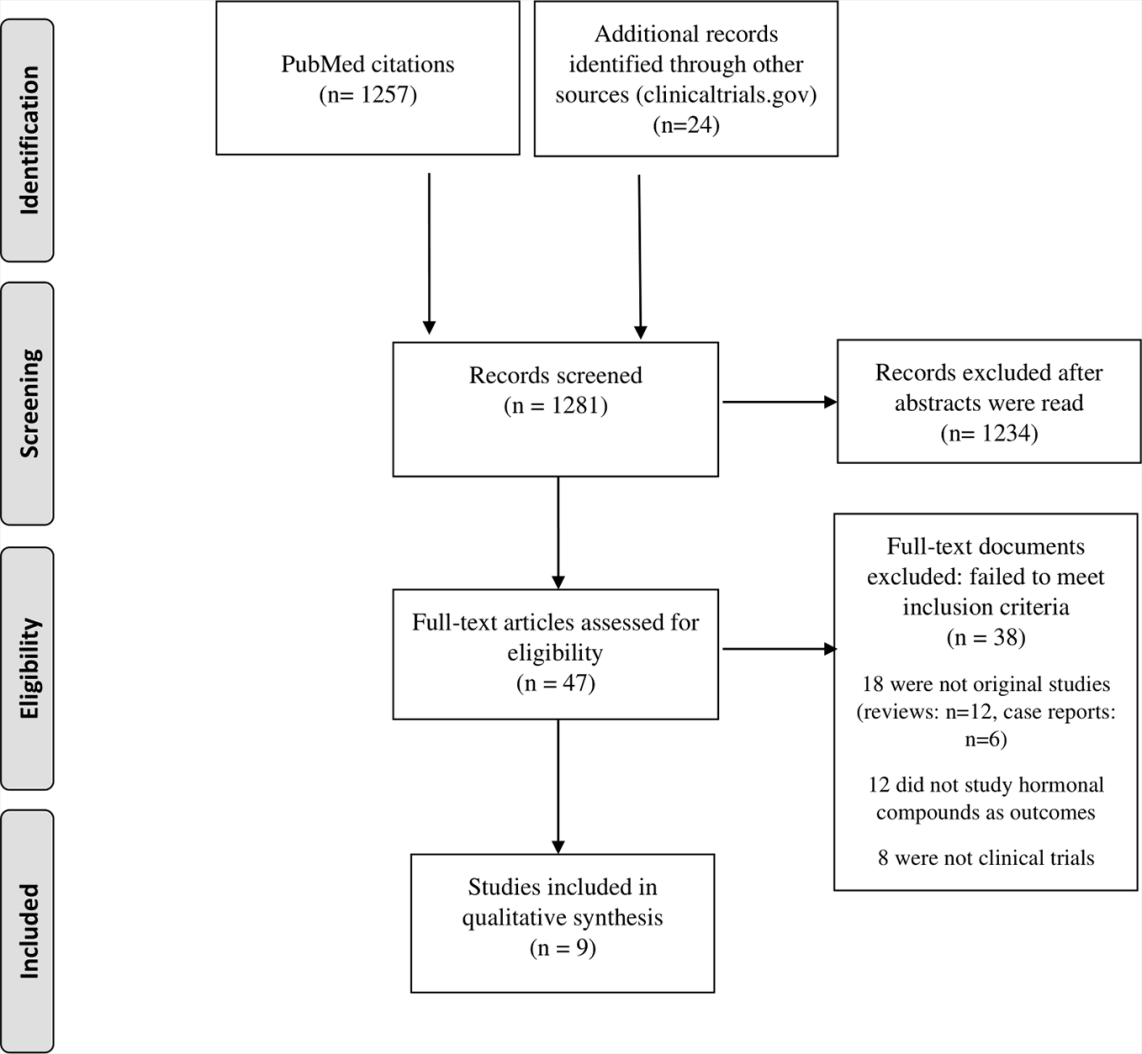
Flowchart for studies inclusion.

A total of 1,281 abstracts were identified, 1,257 in PubMed and 24 through other sources (www.clinicaltrials.gov). Further details of the screening and selection process are described in **Figure 1**.

### Published Articles Reporting Psychopathological Effects of Hormonal Treatments Used for Infertility

Eight trials investigated the effects of hormones used in women undergoing fertility treatments (2, 3, 32–37). All of them evaluated the effect of GnRH antagonists or GnRH agonists on the occurrence of newly diagnosed depressive symptoms in women (**Tables 1**, **2** and **3**). None of them explored the effects of ART on women with a previous history of affective or psychotic disorders or the occurrence of psychotic or manic symptoms in women without a previous diagnosis of mental disorder.

**Table 1:** Main characteristics of published clinical trials reporting psychopathological complications with hormonal treatments for infertility (n=8).

Author and year of publication	Sample size (n)	Country	Mean age (SD) or Median [IQR]	Inclusion criteria	Treatment (main arm and control group)
**Haemmerli Keller et al. (** 32 **)**	119 women	Switzerland	NC-IVF: 34.0 (7.2) c-IVF: 34.9 (4.5)	Women between 18 and 42 years with indication of IVF c-IVF or NC-IVF depending on medical indication and women’s wishes	Non-randomized trial: a) c-IVF (IVF with HMG and GnRH antagonists) (n=62) b) NC-IVF (IVF without gonadotropin stimulation) (n=57)
**Greco et al. (** 33 **)**	236 women	Italy	Modified-NC: 35.2 (3.6) AC: 35.5 (3.8)	Women with indication of IVF at their first IVF cycle	Randomized controlled trial, method of preparation, blastocyst transfer: a) modified natural cycle (n=118) b) artificial protocol (n=118), GnRH agonist combined with oestradiol valerate
**Mamata et al. (** 34 **)**	692 women	India	GnRH antagonists: 30.6 (3.83) GnRH agonists: 30.7 (4.21)	Women from 12 sites in India, aged 18- 45 years undergoing for first cycle of IVF/ICSI	Non-randomized trial: a) Group A - GnRH antagonist (n=232) b) Group B - GnRH agonist (n=460)
**Stenbæk et al. (** 3 **)**	83 women,	Denmark	GnRH antagonist: 31.2 [35.5-28.4] GnRH agonist: 36.4 [37.6-32.7]	Women undergoing first infertility treatment cycle of IVF or ICSI	Randomized trial: a) GnRH antagonist (n=42): daily injections with r-FSH b) GnRH agonist (n=41)
**Bloch et al. (** 2 **)**	108 women	Israel	Total sample: 31.8 (5.4)	Women admitted, period 2006-2007, first or second IVF cycle Age < 42 years No endometriosis No psychopharmacological treatment	Prospective randomized trial: a) Short protocol (GnRH agonist triptorelin plus daily r-FSH (n=60) b) Long protocol (GnRH agonist triptorelin for 14 days, followed by r-FSH (n=48)
**de Klerk et al. (** 36 **)**	391 women	The Netherlands	Mild strategy: 33.0 (3.0) Standard strategy: 32.8 (3.3)	Women who planned IVF, randomization into one protocol No previous history of unsuccessful IVF	Randomized controlled two-center trial: a) Mild ovarian stimulation (GnRH antagonist co-treatment) and single embryo transfer (n=197) b) Standard GnRH agonist long-protocol ovarian stimulation with double embryo transfer (n=194)
**Heijnen et al. (** 35 **)**	404 women	The Netherlands	Mild strategy: 32.9 (3.1) Standard strategy: 32.8 (3.2)	Women who planned IVF or intracytoplasmatic sperm injection with no previous IVF history	Randomized non-inferiority trial: a) GnRH antagonist combined with single embryo transfer (n=205) b) GnRH agonist long protocol and transfer of two embryos (n=199)
**Tapanainen et al. (** 37 **)**	100 women	Finland	Goserelin: 33.6 (4.0) Buserelin: 33.6 (3.8)	Women who planned IVF	Randomized trial: a) Long-acting s.c. goserelin (LHRH agonist) (n=49) b) Buserelin acetate i.n. (LHRH agonist) (n=51) plus 150 IU of HMG/day after 11 days of GnRH-a treatment

AC, artificial cycle; GnRH-a, gonadotrophin-releasing hormone agonist; HADS, Hospital Anxiety and Depression Scale; HMG, human menopausal gonadotrophin; i.n., intranasal; c-IVF, conventional in vitro fertilization; n-IVF, non-conventional in vitro fertilization; IQR, interquartile range; NC, natural cycle; LHRH, luteinizing hormone-releasing hormone; r-FSH, recombinant follicle-stimulating hormone; s.c., subcutaneous; SD, standard deviation.

**Table 2:** Methods and results of published clinical trials reporting psychopathological complications with hormonal treatments for infertility (n=8).

Author and year of publication	Trial objectives	Psychopathological assessment (scales, interviews)	Outcomes	Main results
**Haemmerli Keller et al. (** 32 **)**	To compare the psychological burden of conventional IVF with gonadotropin stimulation and IVF without gonadotropin stimulation	CES-D Brief Symptom Inventory	Mean (pre-post) differences in CES-D between both arms^†^	NC‐IVF patients had significantly lower level of depression than cIVF patients.
**Greco et al. (** 33 **)**	To evaluate clinical pregnancy rates of two methods of endometrial preparation for frozen-thawed single euploid blastocyst transfer: modified natural and artificial cycle with GnRH agonist suppression To compare psychological distress between both strategies	HADS	Mean (pre-post) differences in HADS scores between both arms^†^	No significant differences were found between both groups in anxiety and depression scores before starting treatment, on the days of progesterone administration, at the blastocyst transfer, and at the pregnancy test
**Mamata et al. (** 34 **)**	To understand physical and psychological burden in women under fertility treatment To compared GnRH antagonist protocol with GnRH agonist in terms of physical and psychological burden	HADS HSCL Somatization subscale	Mean (pre-post) differences in HADS scores between both arms^†^	No statistically significant differences were found in physical or psychological burden between the protocols (GnRH antagonist and GnRH agonist)
**Stenbæk et al. (** 3 **)**	To investigate whether women exposed to GnRH agonist protocols exhibit higher levels of mental distress compared to women under GnRH antagonist protocols	Profile of Mood States Perceived Stress Scale Major Depression Inventory	Mean (pre-post) differences in MDI scores between both arms^†^	Although the GnRH antagonist protocol was associated with mood fluctuations during the stimulation phase, mood disturbances were not induced by either of the protocols
**Bloch et al. (** 2 **)**	To determine whether affective symptoms appear with the use of GnRH agonists inducing hypogonadic states during IVF cycles To compare long- (inducing hypogonadism) and short-protocols (without hypogonadism)	Brief Symptom Inventory State-Trait Anxiety Inventory CES-D	Mean (pre-post) differences in CES-D scores between long- and short-protocols^†^	GnRH agonist-induced hypogonadal states were not associated with increased mood symptoms. Both protocols (short and long) were comparable in their effects on the induction of affective symptoms
**de Klerk et al. (** 36 **)**	To compare the impact of unsuccessful IVF on women’s psychological symptoms between mild and standard protocols To compare self-reported symptoms of depression between both protocols	HADS	Mean (SD) on HADS (depression scores) after treatment % of significant depression	At the first IVF treatment cycle, mild ovarian stimulation (including GnRH antagonists) was associated with fewer short-term depressive symptoms than conventional protocols
**Heijnen et al. (** 35 **)**	To investigate whether mild and standard protocols differ in the proportion of term livebirths and women’s well- being, and cost per couple	Assessment (baseline, 1 week after outcomes): HADS HSCL Somatization subscale Subjective Sleep Quality Scale	Differences in HADS scores after each IVF cycle (represented in Figure) between both arms	No statistically significant differences in depressive or anxious symptoms between the protocols
**Tapanainen et al. (** 37 **)**	To investigate successful outcomes of IVF treatment with goserelin depot versus buserelin acetate	Ad hoc questionnaire with a subjective estimation scale of different side effects (e.g., tiredness, depression and irritability) (scores from 1 = absent to 5 = severe)	Mean (SD) scores after treatment^†^	Buserelin group: higher incidence of tiredness, depression, headache and abdominal pain than the goserelin group. No differences between the groups in mental irritability, nausea, or swelling.

^†^Effect sizes for the change (pre-post) in depressive symptoms for each treatment arm could be calculated (see **Table 3**).

CES-D, Center for Epidemiologic Studies Depression Scale; HSCL, Hopkins Symptom Checklist; HADS, Hospital Anxiety and Depression Scale; HMG, human menopausal gonadotrophin (HMG); IVF, in vitro fertilization; NC-IVF, non-conventional in vitro fertilization; SD, standard deviation.

**Table 3:** Effect sizes of changes in depressive symptoms in studies exploring the effect of hormonal treatments for infertility.

Study	Assessment scale	Arm	N	Before treatment		After treatment		Effect size measures	
				Mean	SD	Mean	SD	g	d
									95% CI limits
**Haemmerli Keller et al. (** 32 **)**	CES-D	NC-IVF (no gonadotropin stimulation or very low doses of clomiphene)	Pre (N = 57)	12.7	7.3	13.4	10.9	-0.07	-0.38 to 0.23
			Post (N = 44)						
	CES-D	cIVF (HMG + GnRH antagonist)	Pre (N = 62)	12.2	8.6	15.7	7.9	-0.42	-0.73 to -0.12
			Post (N = 45)						
**Greco et al. (** 33 **)**	HADS-D	Modified-NC (no gonadotropin stimulation; oocyte maturation with hCG)	109	5.3	1.8	6.1	1.1	-0.53	-0.75 to -0.32
	HADS-D	Artificial cycle (GnRH agonist)	113	4.9	1.5	6.8	1.6	-1.22	-1.47 to -0.98
**Mamata et al. (** 34 **)** ^§^	HADS-D	GnRH antagonist	232			-0.1	3.6	-0.03	NA^#^
	HADS-D	GnRH agonist	460			0.1	3.7	0.03	NA^#^
**Stenbæk et al. (** 3 **)** ^†^	MDI	GnRH antagonist	42	8.7	9.6	8.2	7.3	0.06	-0.25 to 0.37
	MDI	GnRH agonist	41	6.3	3.8	7	5.4	-0.15	-0.47 to 0.17
**Bloch et al. (** 2 **)**	CES-D	Long-protocol (GnRH agonist for 14 days+ r-FSH)	48	32.5	7.6	36.2	9.4	-0.43	-0.073 to -0.13
	CES-D	Short- protocol (GnRH agonist + r-FSH from first day of the cycle)	60	30.6	8.5	34.3	9.7	-0.40	-0.67 to -0.14
**Tapanainen et al. (** 37 **)** ^‡^	Subjective depressive symptoms	Goserelin (GnRH agonist)	49	1.11	0.50	1.27	0.55	-0.30	-0.59 to -0.02
	Subjective depressive symptoms	Buserelin (GnRH agonist)	51	1.35	0.87	1.59	1.04	-0.25	-0.53 to 0.03

g, Hedges’ g; d, Cohen’s d; SD, standard deviation; CI, confidence intervals; CES-D, Center for Epidemiologic Studies Depression Scale; cIVF, in vitro fertilization with gonadotropin stimulation; AC, artificial cycle; NC, natural cycle; NC-IVF, in vitro fertilization without gonadotropic stimulation; GnRH, gonadotropin- releasing hormone; HMG, human menopausal gonadotropin; r-FSH, recombinant follicle-stimulating hormone; HADS-D, Hospital Anxiety and Depression Scale—depression subscore; MDI, Major Depression Inventory; NA, not assessed.

^§^In the Mamata et al. (34) study, mean differences in HADS-D were reported. Hedges g’ was calculated taking into account this information.

^†^As median and interquartile range were reported in the study by Stenbæk et al. (3), mean (SD) was computed as suggested by Wan et al.

^‡^In the Tapanainen et al. (37) study, mean (SD) changes in subjective symptoms of depression were obtained from figures with t he procedure described by Labad et al. (28).

^#^d Cohen confidence interval limits were not calculated because means and standard deviations for baseline and final visits were not available.

Haemmerli-Keller et al. (32) carried out a non-randomized trial comparing women undergoing IVF with (cIVF) and without gonadotropin stimulation (NC-IVF) (32). In the cIVF arm (gonadotropin stimulation), human menopausal gonadotropin (HMG) was initiated between days 3 and 5 of the menstrual cycle, and GnRH antagonists were first administered subcutaneously between days 6 and 7 of the menstrual cycle and continued until ovulation induction with urinary human chorionic gonadotropin (hCG). NC-IVF patients received no stimulation at all or very low dosages of clomiphene citrate from day 6 or 7 of the menstrual cycle until the day of ovulation induction with hCG. Depressive symptoms were evaluated in all women by means of the Center for Epidemiologic Studies Depression Scale. The authors found that patients who were given gonadotropin stimulation showed higher rates of depression than women who were not given gonadotropin stimulation.

Greco et al. (33) included 236 women who underwent infertility treatment in a prospective controlled randomized trial (33). Two methods of endometrial preparation for blastocyst transfer were applied: modified natural cycle and artificial cycle. Frozen-thawed single euploid blastocyst transfer by means of a modified natural cycle was applied to 118 women. The hormonal protocol for this treatment arm did not include gonadotropin stimulation and final oocyte maturation was induced with hCG. One hundred 18 women were included in the artificial cycle arm. The hormonal protocol for this treatment arm consisted of the administration of a GnRH agonist (buserelin acetate) combined with oestradiol valerate (33). The authors did not find statistically significant differences between both groups in terms of anxiety and depressive symptoms, neither before the beginning of treatment, nor on the following days after progesterone administration, blastocyst transfer or at the pregnancy test (33), suggesting that the use of GnRH agonists show similar psychological effects compared to blastocyst transfer in a modified natural cycle. However, when calculating the effect sizes for longitudinal changes in depressive symptoms (**Table 3**), both treatment arms were associated with worsening of depressive symptoms, although a greater negative effect was seen in the protocol using GnRH agonists (g = -1.22) when compared to the natural cycle protocol (g = -0.53), that did not include gonadotropin stimulation. It is important to underscore that mean (SD) HADS scores for both groups were below 7, which suggests that depressive symptoms were mild, because a cut-off score ≥ 8 is thought to be appropriate for detecting major depression in the general practice (38).

Stenbaek et al. (3) carried out a randomized trial in a total of 83 women undergoing treatment for infertility (3). Patients were randomized to the GnRH antagonist protocol, which included daily injections with r-FSH treatment, and GnRH agonists, which were administered intranasally. Mood symptoms and neuroticism traits were evaluated by self-reported assessment scales. The authors found that neuroticism was associated with higher scores on psychological distress, independent of the protocol received. Mental distress associated with ART treatments may not be attributed to hypogonadism or any protocol (3). In terms of depressive symptoms, although GnRH agonists seemed to show a slightly poorer profile than GnRH antagonists, effect sizes were very small (**Table 3**), without significant pre-post changes.

Similarly, Mamata et al. (34) carried out a trial across 12 IVF centres in India (34). The authors divided participants into two groups according to the type of treatment they received: a) GnRH antagonists and b) GnRH agonists. Psychopathological symptoms and somatic distress symptoms were assessed by means of the Hospital Anxiety and Depression Scale (HADS) and the Hopkins Symptom Check List (HSCL) during two treatment visits. Although no statistically significant differences in depressive or anxiety symptoms were found between both protocol groups, the percentage of individuals who reported depressive symptoms was higher in those receiving GnRH agonists compared to those under GnRH antagonists, suggesting that women undergoing IVF/ICSI may suffer from higher depressive symptoms than women not undergoing these treatments, irrespective of the protocol assigned.

With the main hypothesis that GnRH agonists may be responsible for the induction of depressive symptoms in women undergoing IVF, Bloch and co-workers (2) carried out a prospective randomized trial with two main treatment groups: a short protocol and a long protocol (2). The long protocol consisted of the subcutaneous administration of the GnRH agonist triptorelin for 14 days. 225 IU of recombinant FSH (r-FSH) were administered in a second step. The short protocol was defined by the administration of the GnRH agonist from the first day of the cycle, followed by concomitant 225 IU r-FSH. The authors hypothesized that a long protocol may induce prolonged hypogonadism compared to a short protocol, a fact that may increase levels of psychological distress and may be associated with higher depressive symptoms (2). The authors found a significant increase in depressive and anxiety symptoms during IVF-ET cycles, which were higher between the hypogonadal phase and the peak in gonadotropin stimulation, at later points in treatment. The hypogonadal state which was GnRH agonist-induced was not found to be associated with increased mood symptoms, suggesting that neither long nor short protocols were correlated with an increase in depression. Effect sizes for the change in depressive symptoms suggest that both protocols are associated mild mood worsening (**Table 3**).

De Klerk et al. (36) investigated the psychopathological effect of IVF treatment on women undergoing mild ovarian stimulation (including GnRH antagonist and single embryo transfer) compared to an standard strategy (long-protocol with GnRH agonist and double embryo transfer) (36). After IVF treatment, women with negative outcome were more likely to present depressive symptoms 1 week after the end of treatment compared to women who received mild IVF treatment. The prevalence of possible depressive disorder (defined as a HADS score>7) was 38.8% for the women in the standard IVF group who underwent multiple IVF cycles against 19.4% of the women in the mild IVF group (p = 0.04). The authors associated these results with the prolonged ovarian suppression by GnRH agonists in those women who underwent the standard IVF, suggesting that these treatments may lead to more symptoms of depression.

Heijnen et al. (35) carried out a randomized, open-label, non-inferiority trial in 404 women with an indication for IVF or ICSI in the Netherlands, of whom 205 received mild ovarian stimulation (single embryo transfer with the administration of GnRH antagonists) and 199 were given standard ovarian stimulation with the transfer of two embryos and with the administration of GnRH agonists (35). The authors assessed depressive symptoms and anxiety by means of the HADS and found no statistically significant differences in psychopathological symptoms between both groups (35).

One of the most relevant studies was the randomized trial carried out by Tapanainen et al. (37). In this IVF study, patients received two types of GnRH agonists. Individuals were randomized to treatment with long-acting subcutaneous goserelin or with intranasally administered buserelin acetate. They both stimulate the production of testosterone and oestrogen in a non-pulsatile manner, which results in the downregulation of both sex hormone systems (37). After 11 days of the administration of either goserelin or buserelin, the administration of 150 IU of HMG/day was started. The authors found that patients receiving buserelin reported more depressive mood, tiredness, and headache 1 week after starting the GnRH agonist, when compared to those treated with goserelin (37). However, when considering the effect sizes for the change in depressive symptoms during the study, both protocols had similar effects (mild worsening) on mood (**Table 3**).

### Non-Published Studies Designed to Assess Psychopathology in Women Receiving Hormonal Treatments for Infertility (Completed or Ongoing)

One trial was registered at clinicaltrials.gov and reported to be completed.

In 2010, Pittman et al. started a double-blind, placebo-controlled, crossover clinical trial in 20 menstruating women with regular menstrual cycles who suffered from unexplained infertility (39). The main goal of this trial was to explore psychopathological symptoms and physical concerns in women receiving clomiphene citrate for superovulation with intrauterine insemination. Women were randomized to treatment with clomiphene citrate or placebo and received this intervention on days 3–7 of their menstrual cycle. Ten patients received clomiphene citrate 50 mg daily and then placebo daily, and 10 women received placebo and then clomiphene citrate daily. Physical, behavioral, and mental symptoms were explored by using the following assessment scales: Follicular Cycle Total Physical Score for the Calendar of Premenstrual Experiences (COPE) self-assessment, the Follicular Cycle Total Behavioral Score for the Calendar of Premenstrual Experiences (COPE) self-assessment, the Luteal Cycle Total Behavioral Score for the Calendar of Premenstrual Experiences (COPE) self-assessment and the Luteal Cycle Total Physical Score for the Calendar of Premenstrual Experiences (COPE) self-assessment. Although not statistically relevant, patients receiving placebo showed higher scores than those receiving clomiphene citrate on the Follicular Cycle Behavioral and Physical Scores (COPE). No other differences in median scores were found in behavioral, mental, and physical symptoms as measured by the luteal scales.

### Quality Assessment and Risk of Bias

The assessment of the risk of bias has been presented in detail in **Table S2**. Of all nine clinical trials included in our systematic review, four were considered to be good (2, 3, 36, 39), four fair (33–35, 37), and only one (32) had poor quality.

We did not identify any trial publication or non-publication of results regarding the occurrence of psychotic symptoms in women undergoing fertility treatments.

## Discussion

In this paper, we carried out a systematic review on available work on the psychopathological effects of hormonal treatments for infertility on women and the effects of these therapies on the occurrence of newly diagnosed mood and psychotic disorders. Finally, nine trials were included, all of them in non-clinical populations. Four trials compared GnRH agonists and GnRH antagonists (3, 34–36), showing a better mood profile (less depressive symptoms) for those hormonal protocols including antagonists in one trial. Two trials compared protocols using either GnRH agonists or GnRH antagonists versus natural cycle protocols that did not use gonadotropin stimulation, reporting a better mood profile in those protocols without gonadotropin stimulation (32, 33). Other two studies comparing long and short protocols of GnRH agonists (2) and two GnRH agonists (buserelin and goserelin) (37) did not find significant differences in mood changes between treatment arms. An unpublished study compared two patterns of clomiphene vs placebo administration with no differences between groups (39).

Most previous published trials evaluated the effect of either GnRH antagonists or GnRH agonists on the occurrence of newly diagnosed depressive disorder or depressive symptoms for the first time in women. None of them investigated the risk or prevalence of relapses in women with a previous diagnosis of depressive disorders or psychotic disorders. When focusing on hormonal compounds other than GnRH agonists or antagonists, one recent trial has been developed to study the effect of clomiphene citrate on women under the IVF protocol (39). Moreover, in the study by Haemmerli Keller et al. (32), patients under the protocol without gonadotropin stimulation could receive low doses of clomiphene (32). This latter study suggests that the lack of gonadotropin stimulation is associated with fewer changes in mood, as the effect size was very small (g = -0.07).

Celano et al. (40) and Wilkins and collaborators (41) pointed out that GnRH agonists (e.g., leuprolide and goserelin) induce hypogonadism (40, 41), which could be related to different psychiatric side effects. In the information about these hormonal drugs (42, 43), depressive symptoms also seem to be reported. These findings have been replicated in subsequent case series (44, 45) and retrospective studies, including the report of a patient treated with leuprolide (46). Furthermore, prophylactic treatment with SSRIs has been reported to prevent the development of depressive symptoms in these patients (45).

In our systematic review, we found that patients receiving ovarian stimulation with GnRH antagonists had increased depressive symptoms compared to patients who did not receive ovarian stimulation (32). One study suggested that treatment with one GnRH agonist, buserelin, had a poorer mood profile than other GnRH agonist (goserelin) 1 week later but showed similar mood changes at the end of the trial (37). However, GnRH agonists did not seem to differ from GnRH antagonists in their associated occurrence of depressive symptoms, suggesting that neither protocol has a psychopathological effect on women undergoing fertility treatments (3, 34–36).

In contrast, Ben Dor et al. (47) studied the effect of GnRH agonists on a sample of 72 healthy women (medication-free; not pregnant; no significant past or current medical illness; reported regular menstrual cycles; and normal physical, gynaecological examinations, and laboratory results) (47). The absence of current or past Axis I psychiatric illness was confirmed by Structured Clinical Interview for DSM-IV. The objective of the study was to better understand whether the acute induction of hypogonadism may determine significant depressive symptoms in healthy premenopausal women, which individual symptoms may be associated with hypogonadism and increase susceptibility to depression and whether changes in plasma levels of ovarian hormones correlate with changes in mood symptoms (47). After a 2-month screening phase, every woman received the first dose of depot leuprolide acetate. Relative to baseline, induced hypogonadism with GnRH agonist was associated with significantly decreased sexual interest, disturbed sleep, and hot flashes but no significant change in any mood-related symptom scores (47). The authors proposed that depressive symptoms associated with GnRH agonist ovarian suppression in menopause could reflect mainly the effects of ovarian suppression on women who are more vulnerable to the development of depression or who are currently depressed (47). However, once again, no clinical trials have explored the effect of leuprolide on women undergoing fertility treatments.

Bloch and collaborators (2) investigated the relationship between affective symptoms and gonadal steroids during *in vitro* fertilization (2). They found that the drop from high oestradiol levels at the oestradiol phase to lower levels at the progesterone phase was correlated with increasing depressive symptoms, suggesting that the abrupt decline in oestrogen levels can precipitate negative mood states. This fact may be responsible for the variability of the presence of depressive symptoms in women undergoing IVF (2). This may be partially in line with some observational studies exploring the prevalence of depressive symptoms in women undergoing fertility treatments. Particularly, Volgsten and co-workers (22) carried out an observational study including couples undergoing IVF or ICSI and applied the Primary Care Evaluation of Mental Disorders (PRIME-MD) system, which is a tool to evaluate the prevalence of psychiatric disorders according to 2% in men, being major depression the most commonly found, irrespective of the hormonal compound they received. More recently, other studies have reported that pregnant women after fertility treatment did not differ in terms of depressive symptoms compared to those naturally pregnant, suggesting that the treatment did not have a negative impact on mood (23). However, the hormonal compounds were not included in the discussion. Evans-Hoeker et al. (48) carried out a cohort study including participants in two previous randomized trials, namely PPCOS II and AMIGOS (48). The first trial compared patients treated with clomiphene citrate versus letrozole, and the second compared gonadotropins, clomiphene citrate and letrozole. Both studies assessed whether maternal depression would have an influence on pregnancy outcomes after non-IVF fertility therapies. They did not find any negative effect of current active depression on non-IVF outcomes. The effect of hormonal compounds used in fertility treatment on the recurrence of major depression have been poorly investigated. Sejbaek et al. (49) carried out a register-based national cohort study including women undergoing IVF, ICSI, and other embryo transfer treatments (49). Women with a previous diagnosis of depression had lower rates of live births. The authors did not find statistically significant differences in rates of depression, but they recommended that women with previous depression may require specific psychiatric attention before starting a new treatment. In the same line, other authors highlight that a history of major depression would be a significant predictor for major depression during fertility treatment (50). Psychosocial support and interventions addressed to women with a previous history of depression have been recommended when initiating infertility treatment.

We did not find any clinical trial specifically investigating the effects of hormonal treatments on depressive symptoms in unipolar or bipolar disorder patients. In a recent prospective observational study that included women receiving infertility treatments with a history of major depressive disorder or bipolar disorder (24), a high risk of depressive relapse was found, even in those women maintaining psychotropic medication. These results suggest that maintenance of medication is not sufficient to avoid affective relapses. In our systematic review, we did not find clinical trials assessing recurrences in patients with affective disorders.

Women with polycystic ovary syndrome (PCOS) show an increased prevalence of higher depression and anxiety scores and increased odds of moderate and severe depressive and anxiety symptoms compared with controls (51). On the other hand, a few studies that have evaluated the impact of PCOS-related treatments (lifestyle interventions and pharmacotherapy) on mood have reported no detrimental effect or even some improvement in depressive and anxiety symptoms and quality of life (51). In addition, clomiphene citrate, a selective oestrogen receptor modulator used to induce ovulation, even in patients with PCOS, has been associated with mood lability and depressed mood in two cross-sectional studies (52, 53). In the cross-sectional, self-report survey of Choi et al. (53), 41% of clomiphene-treated women experienced depressed mood, and 75 of 162 (45%) experienced mood swings during treatment (53). Mood changes (54), suicidal behavior (55), and visual hallucinations (56) have also been associated with clomiphene treatment in case reports.

None of the selected published clinical trials in our systematic review specifically investigated the psychopathological effects of the use of clomiphene citrate in the treatment of infertility on women. In an unpublished randomized double-blind crossover trial in 20 women (53), clomiphene treatment was not associated with mood or behavioral changes, a result that contrasts those of other studies suggesting that clomiphene induces psychological side effects quite frequently (53).

In relation to patients with psychotic disorders, to the best of our knowledge, no clinical trials have evaluated the psychopathological effects of hormonal treatments for infertility on women diagnosed with bipolar disorder, schizophrenia, or other psychotic disorders. A case report linked the use of leuprolide acetate for *in vitro* fertilization (IVF) treatment to a psychotic exacerbation in a 37-year-old woman who suffered from a previous schizoaffective psychosis (57). In this line, a case series of Purvin (58) showed different visual disturbances (some of them persistent) secondary to clomiphene citrate treatment in three women treated for infertility with clomiphene for 4 to 15 months (58). In a review by Seeman (59), there were five case reports of a self-limited psychotic disorder induced by clomiphene (59). All cases were reported to start psychotic symptoms during treatment, to have a paranoid component and to stop when the drug was withdrawn. We did not find any trial reporting the potential effects of hormones used in fertility treatments on women with psychosis or the occurrence of psychotic symptoms in non-diagnosed women. However, some case reports have been reported on the use of clomiphene. A recent national register study compared success rates of fertility treatments in women with and without psychotic disorders prior to the treatment (25), as a part of a cohort of 42,915 Danish women undergoing fertility treatments. Women with previous diagnosis of psychotic disorder had lower rates of success compared to non-psychotic women. The authors did not evaluate depressive symptoms as the main outcomes.

Several limitations in this systematic review should be considered. The most important limitation of this systematic review is the absence of a sufficient number of published articles on the psychopathological effects of hormone compounds used in infertility treatments in women. Furthermore, most clinical trials included in our systematic review excluded patients with a psychiatric history or psychopathological treatment. Therefore, these exclusion criteria might bias the evidence because most studies excluded women with mental illnesses. Although the quality of selected clinical trials was fair to good in most cases (8/9 = 89%), as none of them included patients with serious mental illnesses, the research field needs to conduct more inclusive clinical trials to overcome this limitation.

The prediction of acute exacerbations or the occurrence of newly diagnosed mental disorders is limited by the scarce literature in the field. Furthermore, most studies were focused on depressive or anxiety symptoms, and they did not assess, in general, psychopathological symptoms according to the most commonly used assessment scales for depression. Several authors have reported that psychotic symptoms may occur during fertility treatments, during or after the use of hormone compounds; however, to date, few studies have focused on patients suffering from schizophrenia and other psychotic disorders. Most of them are case reports or case series, and trials are still lacking on this topic. To the best of our knowledge, this is the first systematic review on the psychopathological effects of hormonal treatments for infertility. Although the number of selected clinical trials was low, our systematic review opens new avenues on the investigation of hormone effects in women undergoing infertility treatments. Future studies need to include patients with major mood and psychotic disorders.

## Author Contributions

AG-R and JC conducted the screening and selection processes. AG-R, JC, VS, CG-R, MB, JM and JL contributed to the conceptualization and writing of this manuscript. All authors have approved the final version of the manuscript.

## Funding

This study was supported in part by a grant from the Catalan Agency for the Management of University and Research Grants (AGAUR 2017 SGR 632). The funder had no role in the study design, data collection and analysis, decision to publish, or preparation of the manuscript.

## Conflict of Interest

The authors declare that the research was conducted in the absence of any commercial or financial relationships that could be construed as a potential conflict of interest.

## References

[1] Rooney KL Domar AD The relationship between stress and infertility. Dialogues Clin Neurosci (2018) 20:41–7. 10.31887/DCNS.2018.20.1/klrooney PMC6016043 29946210

[2] Bloch M Azem F Aharonov I Ben Avi I Yagil Y Schreiber S GnRH-agonist induced depressive and anxiety symptoms during in vitro fertilization-embryo transfer cycles. Fertil Steril (2011) 95:307–9. 10.1016/j.fertnstert.2010.07.1073 20801439

[3] Stenbæk DS Toftager M Hjordt LV Jensen PS Holst KK Bryndorf T Mental distress and personality in women undergoing GnRH agonist versus GnRH antagonist protocols for assisted reproductive technology. Hum Reprod (2015) 30:103–10. 10.1093/humrep/deu294 25398970

[4] Stanhiser J Steiner AZ Psychosocial Aspects of Fertility and Assisted Reproductive Technology. Obstet Gynecol Clin North Am (2018) 45:563–74. 10.1016/j.ogc.2018.04.006 30092929

[5] Hanson B Johnstone E Dorais J Silver B Peterson CM Hotaling J Female infertility, infertility-associated diagnoses, and comorbidities: a review. J Assist Reprod Genet (2017) 34:167–77. 10.1007/s10815-016-0836-8 PMC5306404 27817040

[6] Santos C Sobral MP Martins MV Effects of life events on infertility diagnosis: comparison with presumably fertile men and women. J Reprod Infant Psychol (2017) 35:1–13. 10.1080/02646838.2016.1249834 29517288

[7] Briceag I Costache A Purcarea VL Cergan R Dumitru M Briceag I Fallopian tubes–literature review of anatomy and etiology in female infertility. J Med Life (2015) 8:129–31. PMC4392087 25866566

[8] Chambers GM Harrison C Raymer J Petersen Raymer AK Britt H Chapman M Ledger W5, Norman RJ6. Infertility management in women and men attending primary care-patient characteristics, management actions and referrals. Hum Reprod (2019) 34:2173–83. 10.1093/humrep/dez172 31725877

[9] Lunenfeld B Bilger W Longobardi S Alam V D’Hooghe T Sunkara SK The Development of Gonadotropins for Clinical Use in the Treatment of Infertility. Front Endocrinol (Lausanne) (2019) 10:429. 10.3389/fendo.2019.00429 31333582 PMC6616070

[10] Niederberger C Pellicer A Introduction: IVF’s 40th world birthday. Fertil Steril (2018) 110:4. 10.1016/j.fertnstert.2018.05.017 29980260

[11] Bai F Wang DY Fan YJ Qiu J Wang L Dai Y Assisted reproductive technology service availability, efficacy and safety in mainland China: 2016. Hum Reprod (2020) 35:446–52 10.1093/humrep/dez245 32020190

[12] Ray A Shah A Gudi A Homburg R Unexplained infertility: an update and review of practice. Reprod Biomed Online (2012) 24:591–602. 10.1016/j.rbmo.2012.02.021 22503948

[13] Lai Q Zhang H Zhu G Li Y Jin L He L Comparison of the GnRH agonist and antagonist protocol on the same patients in assisted reproduction during controlled ovarian stimulation cycles. Int J Clin Exp Pathol (2013) 6:1903–10. PMC3759499 24040457

[14] Toftager M Bogstad J Bryndorf T Løssl K Roskær J Holland T Risk of severe ovarian hyperstimulation syndrome in GnRH antagonist versus GnRH agonist protocol: RCT including 1050 first IVF/ICSI cycles. Hum Reprod (2016) 31:1253–64. 10.1093/humrep/dew051 27060174

[15] Soria V González-Rodríguez A Huerta-Ramos E Usall J Cobo J Bioque M Targeting hypothalamic-pituitary-adrenal axis hormones and sex steroids for improving cognition in major mood disorders and schizophrenia: a systematic review and narrative synthesis. Psychoneuroendocrinology (2018) 93:8–19. 10.1016/j.psyneuen.2018.04.012 29680774

[16] Rubinow DR Schmidt PJ Sex differences and the neurobiology of affective disorders. Neuropsychopharmacology (2019) 44:111–28. 10.1038/s41386-018-0148-z PMC6235863 30061743

[17] Goldstein JM Lancaster K Longenecker JM Abbs B Holsen LM Cherkerzian S Sex differences, hormones, and fMRI stress response circuitry deficits in psychoses. Psychiatry Res (2015) 232:226–36. 10.1016/j.pscychresns.2015.03.006 PMC4439265 25914141

[18] Holka-Pokorska J Jarema M Wichniak A Clinical determinants of mental disorders occurring during the infertility treatment. Psychiatr Pol (2015) 49:965–82. 10.12740/PP/35958 26688847

[19] Soares CN Zitek B Reproductive hormone sensitivity and risk for depression across the female life cycle: a continuum of vulnerability? J Psychiatry Neurosci (2008) 33:331–43. PMC2440795 18592034

[20] González-Rodríguez A Catalán R Penadés R Bernardo M The oestrogen dysfunction hypothesis in schizophrenia: The need for an integrative approach to treat postmenopausal women. Aust N Z J Psychiatry (2016) 50:1207–8. 10.1177/0004867416649033 27207861

[21] Riecher-Rössler A Oestrogens, prolactin, hypothalamic-pituitary-gonadal axis, and schizophrenic psychoses. Lancet Psychiatry (2017) 4:63–72. 10.1016/S2215-0366(16)30379-0 27856396

[22] Volgsten H Skoog Svanberg A Ekselius L Lundkvist O Sundström Poromaa I Prevalence of psychiatric disorders in infertile women and men undergoing in vitro fertilization treatment. Hum Reprod (2008) 23:2056–63. 10.1093/humrep/den154 PMC2517152 18583334

[23] Salih Joelsson L Tydén T Wanggren K Georgakis MK Stern J Berglund A Anxiety and depression symptoms among sub-fertile women, women pregnant after infertility treatment, and naturally pregnant women. Eur Psychiatry (2017) 45:212–9. 10.1016/j.eurpsy.2017.07.004 28957789

[24] Freeman MP Lee H Savella GM Sosinsky AZ Marfurt SP Murphy SK Predictors of Depressive Relapse in Women Undergoing Infertility Treatment. J Womens Health (Larchmt) (2018) 27:1408–14. 10.1089/jwh.2017.6878 PMC6247370 30067141

[25] Ebdrup NH Assens M Hougaard CO Pinborg A Hageman I Schmidt L Assisted reproductive technology (ART) treatment in women with schizophrenia or related psychotic disorder: a national cohort study. Eur J Obstet Gynecol Reprod Biol (2014) 177:115–20. 10.1016/j.ejogrb.2014.03.013 24721442

[26] Moher D Shamseer L Clarke M Ghersi D Liberati A Petticrew M Preferred reporting items for systematic review and meta-analysis protocols (PRISMA-P) 2015 statement. Syst Rev (2015) 4:1. 10.1186/2046-4053-4-1 25554246 PMC4320440

[27] Wan X Wang W Liu J Tong T Estimating the sample mean and standard deviation from the sample size, median, range and/or interquartile range. BMC Med Res Methodol (2014) 14:135. 10.1186/1471-2288-14-135 25524443 PMC4383202

[28] Labad J Montalvo I González-Rodríguez A García-Rizo C Crespo-Facorro B Monreal JA Pharmacological treatment strategies for lowering prolactin in people with a psychotic disorder and hyperprolactinaemia: a systematic review and meta-analysis. Schiz Res (2020). In press. 10.1016/j.schres.2020.04.031 32507371

[29] Silva V Carvalho A Grande A Martimbianco A Riera R Atallah A Can data extraction from figures perform a meta-analysis? Abstract retrieved from Abstracts of the 20th Cochrane Colloquium. Auckland, New Zealand: Cochrane (2012).

[30] Borenstein M Hedges LV Higgins J Rothstein HR Introduction to Meta-Analysis. John Wiley and Sons (2009). pp. 1–421. 10.1002/9780470743386

[31] Cohen J Statistical Power Analysis for the Behavioral Sciences. New York, NY: Routledge Academic (1988).

[32] Haemmerli Keller K Alder G Loewer L Faeh M Rohner S von Wolff M Treatment-related psychological stress in different in vitro fertilization therapies with and without gonadotropin stimulation. Acta Obstet Gynecol Scand (2018) 97:269–76. 10.1111/aogs.13281 29247514

[33] Greco E Litwicka K Arrivi C Varricchio MT Caragia A Greco A The endometrial preparation for frozen-thawed euploid blastocyst transfer: a prospective randomized trial comparing clinical results from natural modified cycle and exogenous hormone stimulation with GnRH agonist. J Assist Reprod Genet (2016) 33:873–84. 10.1007/s10815-016-0736-y PMC4930788 27221477

[34] Mamata D Ray SK Pratap K Firuza P Birla AR Manish B Impact of different controlled ovarian stimulation protocols on the physical and psychological burdens in women undergoing in vitro fertilization/intra cytoplasmic sperm injection. J Hum Reprod Sci (2015) 8:86–92. 10.4103/0974-1208.158615 26157299 PMC4477455

[35] Heijnen EM Eijkemans MJ De Klerk C Polinder S Beckers NG Klinkert ER A mild treatment strategy for in-vitro fertilisation: a randomised non-inferiority trial. Lancet (2007) 369:743–9. 10.1016/S0140-6736(07)60360-2 17336650

[36] de Klerk C Macklon NS Heijnen EM Eijkemans MJ Fauser BC Passchier J The psychological impact of IVF failure after two or more cycles of IVF with a mild versus standard treatment strategy. Hum Reprod (2007) 22:2554–8. 10.1093/humrep/dem171 17586832

[37] Tapanainen J Hovatta O Juntunen K Martikainen H Ratsula K Tulppala M Subcutaneous goserelin versus intranasal buserelin for pituitary down-regulation in patients undergoing IVF: a randomized comparative study. Hum Reprod (1993) 8:2052–5. 10.1093/oxfordjournals.humrep.a137980 8150902

[38] Olssøn I Mykletun A Dahl AA The Hospital Anxiety and Depression Rating Scale: a cross-sectional study of psychometrics and case finding abilities in general practice. BMC Psychiatry (2005) 5:46. 10.1186/1471-244X-5-46 16351733 PMC1343544

[39] https://clinicaltrials.gov/ct2/show/NCT01291056.

[40] Celano CM Freudenreich O Fernandez-Robles C Stern TA Caro MA Huffman JC Depressogenic effects of medications: a review. Dialogues Clin Neurosci (2011) 13:109–25. 10.31887/DCNS.2011.13.1/ccelano PMC3181967 21485751

[41] Wilkins KM Warnock JK Serrano E Depressive symptoms related to infertility and infertility treatments. Psychiatr Clin N Am (2010) 33:309–21. 10.1016/j.psc.2010.01.009 20385339

[42] Goserelin depot Product Monograph Zoladex® LA Goserelin Depot (10.8 mg Goserelin/depot as goserelin acetate), Luteinizing Hormone - Releasing Hormone Analog (LHRH Analog). (2019). Retrieved from: https://documents.tersera.com/zoladex-ca/10.8mg_ProductMonograph.pdf.

[43] Lupron Depot Center for Drug Evaluation and Research Approval Package for Lupron Depot. (2011). Retrieved from: https://www.accessdata.fda.gov/drugsatfda_docs/nda/2011/020517orig1s025s030s032Review.pdf.

[44] Warnock JK Bundren JC Anxiety and mood disorders associated with gonadotropin-releasing hormone agonist therapy. Psychopharmacol Bull (1997) 33:311–6. 9230649

[45] Warnock JK Bundren JC Morris DW Sertraline in the treatment of depression associated with gonadotropin-releasing hormone agonist therapy. Biol Psychiatry (1998) 43:464–5. 10.1016/S0006-3223(97)00396-X 9532352

[46] Warnock JK Bundren JC Morris DW Depressive symptoms associated with gonadotropin-releasing hormone agonists. Depress Anxiety (1998) 7:171–7. 10.1002/(SICI)1520-6394(1998)7:4<171::AID-DA5>3.0.CO;2-D 9706454

[47] Ben Dor R Harsh VL Fortinsky P Koziol DE Rubinow DR Schmidt PJ Effects of pharmacologically induced hypogonadism on mood and behavior in healthy young women. Am J Psychiatry (2013) 170:426–33. 10.1176/appi.ajp.2012.12010117 PMC3952563 23545794

[48] Evans-Hoeker EA Eisenberg E Diamond MP Legro RS Alvero R Coutifaris C Major depression, antidepressant use, and male and female fertility. Fertil Steril (2018) 109:879–87. 10.1016/j.fertnstert.2018.01.029 PMC5973807 29778387

[49] Sejbaek CS Hageman I Pinborg A Hougaard CO Schmidt L Incidence of depression and influence of depression on the number of treatment cycles and births in a national cohort of 42,880 women treated with ART. Hum Reprod (2013) 28:1100–9. 10.1093/humrep/des442 23300199

[50] Holley SR Pasch LA Bleil ME Gregorich S Katz PK Adler NE Prevalence and predictors of major depressive disorder for fertility treatment patients and their partners. Fertil Steril (2015) 103:1332–9. 10.1016/j.fertnstert.2015.02.018 PMC4417384 25796319

[51] Dokras A Stener-Victorin E Yildiz BO Li R Ottey S Shah D Androgen Excess- Polycystic Ovary Syndrome Society: position statement on depression, anxiety, quality of life, and eating disorders in polycystic ovary syndrome. Fertil Steril (2018) 109:888–99. 10.1016/j.fertnstert.2018.01.038 29778388

[52] Blenner JL Clomiphene-induced mood swings. J Obstet Gynecol Neonatal Nurs (1991) 20:321–7. 10.1111/j.1552-6909.1991.tb01695.x 1941294

[53] Choi SH Shapiro H Robinson GE Irvine J Neuman J Rosen B Psychological side-effects of clomiphene citrate and human menopausal gonadotrophin. J Psychosom Obstet Gynaecol (2005) 26:93–100. 10.1080/01443610400022983 16050534

[54] Aussedat M Jean-Louis J Djahangirian O Brochet MS Clomiphene for the Treatment of Male Infertility: A Case Report of Mood Change and a Literature Overview. Curr Drug Saf (2017) 12:208–15. 10.2174/1574886312666170616092036 28625145

[55] Knight JC Pandit AS Rich AM Trevisani GT Rabinowitz T Clomiphene-Associated Suicide Behavior in a Man Treated for Hypogonadism: Case Report and Review of The Literature. Psychosomatics (2015) 56:598–602. 10.1016/j.psym.2015.06.003 26362915

[56] Venkatesh R Gujral GS Gurav P Tibrewal S Mathur U Clomiphene citrate-induced visual hallucinations: a case report. J Med Case Rep (2017) 11:60. 10.1186/s13256-017-1228-0 28260524 PMC5338088

[57] Abu-Tair F Strowitzki T Bergemann N Exacerbation of a schizoaffective psychosis after in vitro fertilization with leuproreline acetate. Nervenarzt (2007) 78:691–2. 10.1007/s00115-007-2276-2 17487467

[58] Purvin VA Visual disturbance secondary to clomiphene citrate. Arch Ophthalmol (1995) 113:482–4. 10.1001/archopht.1995.01100040102034 7710399

[59] Seeman M Transient psychosis in women on clomiphene, bromocriptine, domperidone and related endocrine drugs. Gynecol Endocrinol (2015) 31:751–4. 10.3109/09513590.2015.1060957 26291819

