# Supplementary material for: Women Undergoing Hormonal Treatments for Infertility: A Systematic Review on Psychopathology and Newly Diagnosed Mood and Psychotic Disorders
Source: Front Psychiatry. 2020 May 26;11:479. doi: 10.3389/fpsyt.2020.00479 (PMC7264258; doi:10.3389/fpsyt.2020.00479)
Supplement: Supplementary file 1 [file Table_1.pdf]

Table S1. PICO scheme of the systematic review.

Patient, Population or Problem	Intervention	Comparison	Outcome
What are the characteristics of the patients or population?	***What interventions are we considering?***	***What is the alternative to the intervention?***	***What are the relevant outcomes?***
Healthy individuals or patients with major mood disorders (major depression, bipolar disorder), schizophrenia or other psychotic disorders.	Hormonal treatments for infertility.	Any comparator (placebo o other hormonal protocol of infertility treatment)	Changes in mood or psychotic symptoms.<br><br>Newly diagnosed major depression or relapses in major psychiatric illnesses (major depression, bipolar disorder, schizophrenia).
