# Supplementary material for: Women Undergoing Hormonal Treatments for Infertility: A Systematic Review on Psychopathology and Newly Diagnosed Mood and Psychotic Disorders
Source: Front Psychiatry. 2020 May 26;11:479. doi: 10.3389/fpsyt.2020.00479 (PMC7264258; doi:10.3389/fpsyt.2020.00479)
Supplement: Supplementary file 2 [file Table_2.pdf]

Table S2. Risk of bias summary of clinical trials included in our systematic review.

Bias	Haemmerli Keller et al., 2018 (32)	Greco et al., 2016 (33)	Mamata et al., 2015 (34)	Stenbæk et al., 2015 (3)	Bloch et al., 2011 (2)	de Klerk et al., 2007(36)	Heijnen et al., 2007 (35)	Tapanainen et al., 1993 (37)	NCT01291056 (39)
Random sequence generation	high	low	low	low	low	low	low	low	low
Allocation concealment	unclear	low	unclear	low	low	low	low	low	low
Blinding of participants and researchers	high	high	high	low	low	low	high	unclear	low
Blinding of outcome assessment	low	low	low	low	low	low	low	unclear	low
Incomplete outcome data	low	low	low	low	low	low	low	low	low
Selective reporting	low	low	low	low	low	low	low	low	low
Other bias	low	unclear	low	low	low	low	low	low	low
**Quality of the clinical trial**	poor	fair	fair	good	good	good	fair	fair	good
