# Supplementary material for: Women Undergoing Hormonal Treatments for Infertility: A Systematic Review on Psychopathology and Newly Diagnosed Mood and Psychotic Disorders
Source: Front Psychiatry. 2020 May 26;11:479. doi: 10.3389/fpsyt.2020.00479 (PMC7264258; doi:10.3389/fpsyt.2020.00479)
Supplement: Supplementary file 3 [file Table_3.pdf]

Table S3. Confidence intervals for effect sizes (Cohen's d) of clinical trials with available information. Sensitivity analyses with different estimated correlations for pre-post depressive scores.

Study	Arm	N	Effect sizes (Cohen's d 95% confidence limits) calculated with different estimated correlation coefficients			
			r= 0	r= 0.25	r= 0.5	r= 0.75
**Haemmerli Keller et al., 2018 (32)**	NC-IVF (no gonadotropin stimulation or very low doses of clomiphene)	Pre (N= 57) Post (N= 44)	-0.49 to 0.34	-0.44 to 0.29	-0.38 to 0.23	-0.30 to 0.16
	cIVF (HMG + GnRH antagonist)	Pre (N= 62) Post (N= 45)	-0.84 to 0.001	-0.79 to -0.05	-0.73 to -0.12	-0.65 to -0.20
**Greco et al., 2016 (33)**	Modified-NC (no gonadotropin stimulation; oocyte maturation with hCG)	109	-0.81 to -0.26	-0.80 to -0.29	-0.75 to -0.32	-0.70 to 0.37
	Artificial cycle (GnRH agonist)	113	-1.53 to -0.92	-1.50 to -0.95	-1.47 to -0.98	-1.43 to -1.02
**Stenbæk et al., 2015 (3)**	GnRH antagonist	42	-0.37 to 0.49	-0.31 to 0.43	-0.25 to 0.37	-0.17 to 0.28
	GnRH agonist	41	-0.58 to 0.29	-0.53 to 0.23	-0.47 to 0.17	-0.39 to 0.09
**Bloch et al., 2011 (2)**	Long-protocol (GnRH agonist for 14 days + r-FSH)	48	-0.84 to 0.02	-0.79 to -0.07	-0.073 to -0.13	-0.66 to -0.21
	Short- protocol (GnRH agonist + r-FSH from first day of the cycle)	60	-0.77 to -0.04	-0.72 to -0.08	-0.67 to -0.14	-0.060 to -0.21
**Tapanainen et al., 1993 (37)**	Goserelin (GnRH agonist)	49	-0.070 to 0.10	-0.65 to 0.05	-0.59 to -0.02	-0.51 to -0.09
	Buserelin (GnRH agonist)	51	-0.64 to 0.14	-0.59 to 0.09	-0.53 to 0.03	-0.45 to -0.04

Abbreviations: cIVF, In vitro fertilization with gonadotropin stimulation; AC, artificial cycle; NC, natural cycle; NC-IVF, in vitro fertilization without gonadotropic stimulation; GnRH, gonadotropin-releasing hormone; HMG= Human menopausal gonadotropin; r-FSH= recombinant follicle-stimulating hormone.
